# Plague Prevention and Therapy: Perspectives on Current and Future Strategies

**DOI:** 10.3390/biomedicines9101421

**Published:** 2021-10-09

**Authors:** Raysa Rosario-Acevedo, Sergei S. Biryukov, Joel A. Bozue, Christopher K. Cote

**Affiliations:** Bacteriology Division, United States Army Medical Research Institute of Infectious Diseases (USAMRIID), Fort Detrick, MD 21702, USA; raysa.rosarioacevedo.mil@mail.mil (R.R.-A.); sergei.s.biryukov.civ@mail.mil (S.S.B.); joel.a.bozue.civ@mail.mil (J.A.B.)

**Keywords:** *Yersinia pestis*, plague, antibiotic, vaccine, antibodies

## Abstract

Plague, caused by the bacterial pathogen *Yersinia pestis*, is a vector-borne disease that has caused millions of human deaths over several centuries. Presently, human plague infections continue throughout the world. Transmission from one host to another relies mainly on infected flea bites, which can cause enlarged lymph nodes called buboes, followed by septicemic dissemination of the pathogen. Additionally, droplet inhalation after close contact with infected mammals can result in primary pneumonic plague. Here, we review research advances in the areas of vaccines and therapeutics for plague in context of *Y. pestis* virulence factors and disease pathogenesis. Plague continues to be both a public health threat and a biodefense concern and we highlight research that is important for infection mitigation and disease treatment.

## 1. Plague History and Disease Presentation

*Yersinia pestis* is a Gram-negative coccobacillus that is the etiologic agent of plague, an acute infectious disease that has played an important role in human history [1,2,3,4,5]. This bacterium has been responsible for three devastating pandemics that led to millions of deaths [6,7,8]. The first pandemic, referred to as the “Justinian Plague”, occurred in 541 AD, originated in Central Africa, and spread to the Mediterranean region [7]. The “Black Death” of 1347 was the second major pandemic and occurred during the 14th century, originating in Central Asia and eventually reaching the Middle East, North Africa, and Europe. The third pandemic took place in 1855 in Yunnan, China and spread globally via infected rats on ships likely originating from Hong Kong [7,9,10]. This bacterium has impacted human civilization throughout history and continues to be of concern [5].

Dissemination of *Y. pestis* among wild rodents and humans takes place in two different cycles, enzootic and epizootic [11]. Enzootic refers to when the infection, at low levels, cycles naturally among wild rodents (e.g., rats, squirrels, marmots, and prairie dogs). Sporadically, other susceptible species can be infected, causing an outbreak—this is referred to as epizootic plague. In this disease cycle, animals become infected and ultimately die as fleas from infected natural reservoir species begin to seek other blood sources and the probability of human transmission is increased [11].

**Figure 1:**
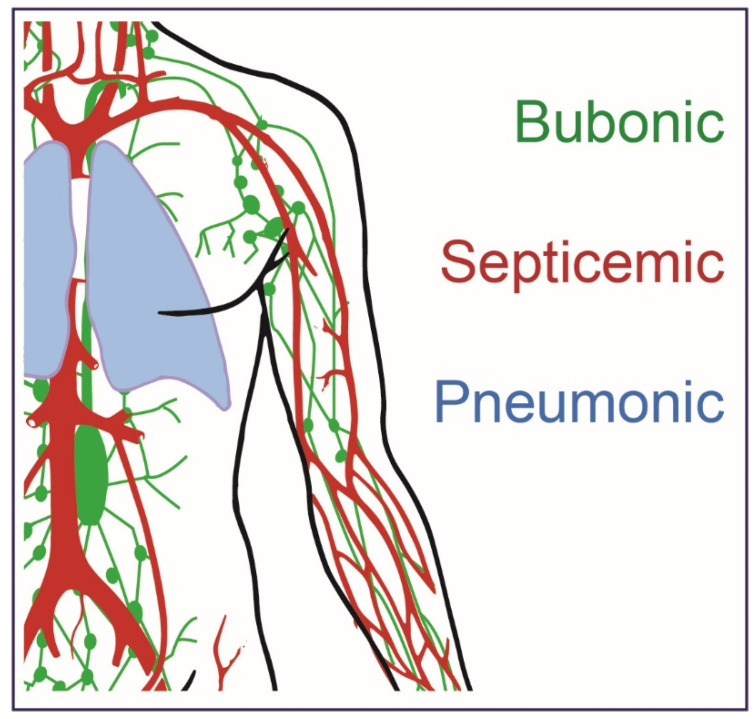
*Y. pestis* infection can lead to three forms of plague. Bubonic plague can result from flea bites or handling infected animals and can result in a severe localized infected lymph node referred to as a buboe (green). Septicemic plague can result as a secondary issue arising from bubonic disease or if the bacteria are introduced directly to the blood stream (red). Pneumonic plague is the most severe form of the disease and can result in person-to-person spread if untreated and initiated by the deposition of *Y. pestis* bacteria into the respiratory tract of individuals (blue).

*Y. pestis* can cause three forms of plague disease: bubonic, septicemic, and pneumonic (Figure 1). Historically, the bubonic form of plague has been the most common and can be transmitted to humans by a bite from an infected flea or by handling an animal infected with *Y. pestis* [12,13,14,15]. After transmission, the *Y. pestis* bacteria disseminate to the nearest lymph node where they colonize and subsequently proliferate. The incubation time ranges from approximately 2–8 days and during that period patients will experience flu-like symptoms, such as chills, fever and headaches [16,17,18,19]. As the bacteria continue to multiply, the lymph nodes become swollen and extremely painful. Infected lymph nodes (referred to as buboes when attributable to plague) are generally found in specific areas of the body, such as the neck, under the chin, armpits and groin. If left untreated, these buboes can become necrotic and cause hemorrhaging ultimately leading to lethal disease in approximately 50–60% of cases [20]. A culture isolated from a blood sample or fluid from the swollen lymph node may be necessary for the diagnosis and identification of *Y. pestis* [17]. Several classes of antibiotics are effective in treating bubonic plague and if treatment is initiated promptly, antibiotics can significantly increase survival rates [19,21,22]. Aminoglycosides, such as streptomycin and gentamicin, tetracyclines, and the fluoroquinolone ciprofloxacin are some effective antibiotics for bubonic plague treatment [20,23,24].

In other more severe cases, the bacteria can enter the blood stream directly and multiply, causing septicemic plague. The increased number of bacteria in the blood causes the release of endotoxins, leading to ischemic necrosis. In those cases, *Y. pestis* directly infects the blood without developing bubonic plague symptoms (resulting in primary septicemic plague) [25]. Furthermore, septicemia may induce intravascular coagulation that may lead to gangrene of the extremities. The prognosis for these patients is poor, with mortality rates reaching 100% in untreated individuals. Even when the appropriate treatment is administered, mortality rates remain high [26]

A third manifestation of plague, pneumonic plague, can be transmissible either person-to-person or by secondary pneumonia, resulting from the colonization of the lungs with *Y. pestis* via hematogenous spreading [1,27]. If droplets containing plague bacteria are inhaled, the result can be primary pneumonic plague. Secondary pneumonic plague may be associated with primary septicemic plague or as a complication of bubonic plague. In the United States, approximately 5–10% of patients develop secondary pneumonic plague [28]. Symptoms of plague include chills, fever, body pains, headache, weakness, dizziness, and chest discomfort [29]. These rather non-descript symptoms mirror many other infectious diseases, which can lead to frequent misdiagnosis and subsequent poor disease outcomes for the patients. Blood cultures or a sputum sample can be used to diagnose pneumonic plague [30]. Pneumonic plague is considered highly contagious and is nearly 100% lethal if appropriate treatment is not administered within the first 20 h after onset of symptoms [30].

Due to rapid spread, high fatality rates, and the ability to transmit via aerosols, *Y. pestis* has been classified as a Tier 1 select agent by the United States Department of Health and Human Services and is considered an agent of biothreat concern [31,32]. Numerous studies have been performed in mice and other species in order to elucidate *Y. pestis* pathogenesis and pneumonic plague disease progression in animal models of human disease [25,33,34,35]. It has been reported that during the first 36 h post infection in mice, there is an increase in bacterial replication in the lungs with no appreciable changes in cytokines or chemokines levels [36,37]. In this pre-inflammatory phase, it has been shown that *Y. pestis* suppresses the host immune response in order to facilitate pulmonary infection [38,39]. After 36 h, the pro-inflammatory phase begins, where there is an upregulation of cytokine or chemokine levels, resulting in a critical point that can lead to death [40].

## 2. *Yersinia pestis* Virulence Plasmids

*Y. pestis* must be able to survive in and infect very diverse hosts, including both fleas and mammals. There are numerous essential virulence factors and effector proteins that are either encoded on the chromosome of *Y. pestis* or carried on its plasmids. It is believed that *Y. pestis* evolved from the genetically related Yersinia species *Yersinia pseudotuberculosis* (usually an intestinal pathogen) through the process of gaining plasmids, most likely several thousands of years ago [4,41,42].

*Y. pestis* carries three plasmids, two of which are unique to this species: pMT1 (or pFra), which encodes the F1 capsular antigen and pPCP (or pPst or pPla), which carries the gene for the virulence factor plasminogen activator. The third plasmid is common to the human pathogenic Yersiniae and is known as pCD1 (calcium dependence), pYV (Yersinia virulence), or pLcr (low calcium response). This plasmid is responsible for the synthesis of a number of anti-host factors and is an absolute requirement for virulence.

The pMT1/pFra plasmid (100–110 kb) carries two important *Y. pestis* virulence factors involved with survival/infection of these two different hosts: the murine toxin Ymt and the F1 capsular protein. Ymt is a phospholipase D which has toxic properties for mice and rats but not for other animals [43,44,45]. More importantly, Ymt is required for survival within the midgut of the flea by protecting it against some components of plasma [46]. In addition, the *caf* operon encoding for the F1 capsular antigen protein (referred to here as F1) is also located on pMT1. The F1 antigen has been described both as capsular and fimbrial-like as it is composed of fibers [47,48,49,50]. The F1 protein is produced abundantly in vivo during infection of a mammal and in vitro when grown at 37 °C. F1 is thought to protect *Y. pestis* from uptake by host phagocytic cells [51]. However, naturally derived and genetically engineered F1-negative *Y. pestis* strains have been described and remain virulent in animal models of plague [52,53].

The other *Y. pestis* specific plasmid (pPCP/pPst/pPla) is 9.5 kb and carries genes that encode for pesticin, pesticin immunity protein, and plasminogen activator (Pla) [54]. Pla appears to be a multifunctional protein and belongs to the omptin family of enterobacterial surface proteins. One of the functions of Pla during infection is to cleave numerous host substrates, such as those involved in coagulation and fibrinolysis [55]. During bubonic plague, Pla activates host fibrolysis to allow bacterial dissemination from the site of infection [56,57]. Furthermore, this protein is believed to alter the host immune response during pneumonic plague to allow outgrowth of the bacteria within the lungs [58,59]. In addition to its ability to cleave proteins, it has a role in mediating bacterial adhesion to host cells and extracellular matrices [60,61,62,63,64].

The final plasmid is pCD1/pYV/pLcr (68–75 kb), which is common to all human pathogenic Yersiniae, is essential for virulence, and carries genes encoding for the Type III secretion system (T3SS) and effector proteins known as Yops (Yersinia outer protein). The T3SS forms a syringe-like structure (injectisome) made up of 27 proteins, including the LcrV protein, which is both a structural and effector protein and is critical for virulence [65,66,67,68,69,70,71,72,73,74,75,76]. The injectisome is able to “inject” host cells when in close contact with Yops. Two of the Yops (YopB and YopD) function to transport the other six effector proteins into the host cell (YopO, YopH, YopM, YopT, YopJ, and YopE). Toxic activities of the Yops include disruption of the cytoskeleton, interference with phagocytic activity, prevention of proinflammatory cytokine synthesis, inhibition of the oxidative burst, and induction of programmed cell death (apoptosis). The overall effect of the Yops is to block phagocytic uptake of the bacteria by host macrophages and polymorphonuclear leukocytes [77,78,79,80,81,82].

## 3. Outbreak Prevention

Despite the efforts of public health agencies to monitor and prevent this disease, reports of plague outbreaks continue to exist in various parts of the word [83,84,85]. The United States, China, India, Vietnam, Democratic Republic of the Congo, Peru, and Madagascar are among the countries that have confirmed plague cases annually and their respective climates, public health infrastructure, surveillance programs, and socio-economic differences make outbreak prevention strategies and severity of outbreaks unique in each case [86,87,88]. Madagascar is currently the most affected country with the highest numbers of recently reported cases of plague, representing approximately 75% of the reported cases in the world [87,89,90]. Differences in weather and temperature patterns affect the abundance of fleas [89,91] which in turn influences the start of annual transmission that generally arises between the months of September to April in Madagascar [92]. Given the right conditions, it may be possible for person-to-person transmission mediated by human ectoparasites—especially in underdeveloped but densely populated areas [93]. A total of 2417 suspect plague cases, including 209 deaths, were reported by the Word Health Organization (WHO) from August to November in 2017 in Madagascar [94]. Generally, bubonic plague cases in Madagascar are commonly concentrated in rural areas, while pneumonic cases are more prevalent in urban locations [95]. In 2017, the majority (77%; 1854 total) reported were clinically classified as pneumonic plague whereas 15% (355) were classified as bubonic plague. Only one of the reported cases was diagnosed as septicemic plague while the remaining 207 cases remain unclassified [94]. Control and prevention of plague cases in Madagascar has proven to be challenging. Most of the prevention strategies are focused on the rodent host and the flea vectors [91]. Inappropriate use of chemical insecticides inadvertently selects for flea resistance, which leads to the survival of *Y. pestis*-infected fleas [91]. Food handling/storage procedures, community sanitation standards, and environmental factors of the household and its surroundings are parameters that can increase human interactions with rodent reservoirs [96]. Importantly, distribution of supplies and equipment including hand washing facilities, sanitation standards, disinfectants (e.g., laundry soap, chlorine powder, disinfectant sprays), and personal protective equipment contribute to an efficient plague prevention strategy [94].

## 4. Current Antibiotic Therapies

Due to the rapid onset and rather non-descript disease characteristics of plague, the key to positive patient outcomes, particularly after infection with aerosolized bacteria, is early diagnosis and rapid implementation of appropriate medical countermeasures. The gold-standard for plague diagnostics continues to be culture-positive samples (normally blood or sputum samples) but other molecular diagnostic assays have been used or continue to be developed [2].

If diagnosed accurately and early after infection, human plague cases can be controlled by the appropriate administration of antimicrobial drugs, including aminoglycoside, tetracyclines, fluroquinolones, and sulfonamides [91,97,98,99,100,101]. Generally, successful treatment of *Y. pestis* infections requires early recognition and the administration of an effective antibiotic during the first 24 h after the onset of symptoms that often present within 24 to 48 h post-infection [18]. According to the United States Centers for Disease Control and Prevention (CDC), the majority of human plague cases can be treated successfully with antibiotics [18]. The most effective antibiotics against *Y. pestis* are aminoglycosides, such as streptomycin and gentamicin, and accordingly, streptomycin is the first-line antibiotic for treatment of plague in most cases; both can be administered and are recommended for all adults, including pregnant women, immunocompromised patients, and children (although reduced dosages may be warranted in some cases) [102]. Chloramphenicol is another suitable agent for bubonic or septicemic plague and can be administered in conjunction with aminoglycosides [18,102]. Doxycycline and tetracycline are acceptable alternate antimicrobial agents as primary treatment for patients with uncomplicated plague and can also be used in conjunction with other antibiotics or in patients intolerant of aminoglycosides [18]. Fluoroquinolones, such as ciprofloxacin, have been demonstrated to have pharmacokinetic properties that make them suitable for plague therapy. Ciprofloxacin possesses bactericidal activity and its efficacy has been tested in vitro, in vivo, and in patients with bubonic plague [103,104]. Individuals in close contact with people afflicted with pneumonic plague, exposed to *Y. pestis* infected fleas, or who have been handling body fluids or tissues infected with *Y. pestis* should receive prophylactic antibiotic therapy.

The emergence of multidrug-resistant (MDR) strains of bacterial pathogens, including *Y. pestis*, is one of the most critical issues facing public health due to the difficulty in treatment, the high cost associated with medical care (particularly in developing countries) and increased mortality rates associated with the drug-resistant phenotypes [105]. This increased incidence of antibiotic resistance is likely due to the close proximity between humans and rodents, the extensive use of antibiotics in animal husbandry, and/or the presence of antibiotics in contaminated hospital waste. In addition to being the most recent epicenter of plague infections, Madagascar is also a focus for MDR *Y. pestis* strains. In 1995, two different strains of naturally occurring antibiotic-resistant *Y. pestis* were isolated in Madagascar. The first one, *Y. pestis* 17/95 was isolated from a 16-year-old boy, and the second strain 16/95 was isolated from a 14-year-old boy; both cases were diagnosed with bubonic plague [97,106,107]. Galimand et al. determined that strain 17/95 isolated from the city of Ambalavao was resistant to eight antimicrobial agents, including those for therapy and prophylaxis—while strain 16/95, isolated from the city of Ambohimahasoa, demonstrated high levels of streptomycin-resistance [97,106]. The multiple antibiotic resistance genes in both MDR strains of *Y. pestis* were all located on plasmids belonging to different incompatibility (Inc) groups [108]. *Y. pestis* strain 17/95 encoded MDR genes on pIP1202 in the Inc6-C group of plasmids, while *Y. pestis* strain 16/95 harbored MDR genes on pIP1203, which belongs to the IncP group of plasmids [97]. In 1998, a novel doxycycline-resistant strain was isolated from the spleen of a rat (*Rattus norvegicus*) in Antananavo, Madagascar [109]. To date, these characterized MDR *Y. pestis* strains were isolated from distinct hosts, at different times, and from distant locations [109]. In addition, Cabanel, et al., 2018 cited that independent horizontal transfer may be the reason for unrelated plasmids among these MDR strains. Other drug-resistant strains and antimicrobial resistance mechanisms have been reported and all aspects of drug-resistance in *Y. pestis* must remain an important research priority [110,111].

## 5. Vaccines

Due to the relative ease of transmissibility, rapid course of disease, non-descript clinical signs and symptoms, high mortality, and antibiotic resistance potential, effective vaccine strategies are needed. An effective and safe plague vaccine is important from a public health perspective but also in context of national biodefense strategies [2,33,112,113]. The first plague vaccines, developed late in the 19th century, consisted of killed whole cells of *Y. pestis* [114].

An immunogenic and somewhat less-reactogenic licensed vaccine (USP) containing a formalin-killed highly virulent 195/P strain of *Y. pestis*, was effective in preventing or ameliorating bubonic disease, as seen by the low incidence of plague cases in military personnel serving in Vietnam. However, the extent of efficacy remains in question, and it is thought that the protection was almost entirely based upon titers to the F1 capsular antigen [115,116,117,118]. In vivo data suggested that this vaccine might not offer optimal protection against pneumonic plague [119,120,121,122,123,124,125]. Such vaccines may not protect against genetically engineered or naturally occurring F1-negative strains, which often maintain significant virulence despite the loss of capsule. The killed whole-cell preparation vaccines, in the absence of frequent boosting, failed to provide long-term protection against bubonic plague.

Live bacterial vaccines are protective, and in many cases, contain a nearly complete array of native antigens—thereby reducing the chances of breakthrough infections. However, live attenuated vaccines are also considered more reactogenic than other vaccination approaches, and may cause safety concerns in certain subsections of the community (e.g., elderly or immune-compromised), and depending upon the vaccination strategy may elicit only short-term immunity [114]. Candidate live vaccines have included recombinant *Y. pestis*, *Y. pseudotuberculosis*, and *Salmonella* strains [126,127,128].

Live attenuated *Y. pestis* vaccines, such as the EV strain (derived and attenuated in the 1920s by serial passaging a virulent *Y. pestis* parent strain isolated from a patient identified as EV), have been in use in various parts of the world for decades [129]. Evidence demonstrated that these live vaccine strategies were able to protect against pneumonic and bubonic plague and induced high antibody titers [119,130]. Unfortunately, these vaccines may have severe side effects and systemic reactions in non-human primates [119] and humans [131], and only induce short-lived protection that require annual boosters [132,133]. 

Recently, additional live attenuated vaccine strategies using attenuated *Y. pestis* strains are also being developed and their corresponding immune responses evaluated [134,135]. Bubeck and Dube achieved significant protection when using a Δ*yopH* live vaccine strain [136] and Bozue et al., demonstrated that a *Y. pestis* Δ*yscN* mutant strain also offered significant protection against challenge with a fully virulent strain of *Y. pestis* [134,137]. Follow-on studies further characterized the vaccine potential of Δ*yscN* strains of *Y. pestis* as well as a strain lacking both the pigmentation (*pgm*) locus and the pPst virulence plasmid [138,139,140,141]. In addition, promising results were obtained with *Y. pseudotuberculosis* based live vaccines that conferred protection and immunological memory [142,143,144,145].

**Figure 2:**
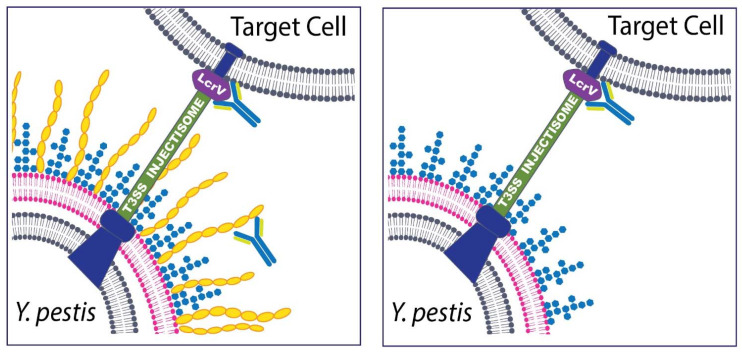
Schematic of Antibodies that target *Y. pestis* and are effective at ameliorating disease. *Y. pestis* is a Gram-negative bacterium that expresses multiple potential targets that have been exploited for the development of novel medical countermeasures. Antibodies generated by active vaccination or administered via passive immunization have been successful at protecting cell culture and animals from *Y. pestis* infection. These targets include The F1 capsular antigen (yellow structures depicted in the left panel) and the LcrV (V antigen) component of the T3SS injectisome. The capsule, while not absolutely necessary for virulence, has been the target of both vaccine and therapeutic strategies. The LcrV protein has been shown to be essential for virulence and has been a successful target in both active and passive immunization studies and is often combined with the F1 capsular antigen (i.e., the F1-V vaccine or mAb cocktail experiments). (Left Panel) In most cases, the *Y. pestis* bacterium will interact directly with host target cells via the T3SS injectisome and a robust anti-phagocytic capsule will also be present; thus, both anti-F1 and anti-V antibodies are potentially effective. (Right Panel) Non-encapsulated strains of *Y. pestis* can be found in nature or engineered in the laboratory. In cases of infection caused by non-encapsulated *Y. pestis*, the antibodies to the F1 capsular antigen are no longer effective and the protective immune response generated by a vaccine or the effective therapeutic mAb relies solely on the anti-V antibodies. A lipopolysaccharide structure (LPS) often observed in *Y. pestis* grown at 37 °C is depicted in blue in both panels. Other protective antigens have been identified and other novel antigen targets will almost certainly be identified in ongoing research efforts.

Besides whole-cell based vaccines, considerable work on subunit vaccine strategies has been completed. With recombinant DNA technology and improved protein chemistry, antigens can be purified for subunit vaccine development. Protein-based subunit vaccines have the potential of increased stability and consistency between vaccine preparations along with reduction in adverse effects that are often correlated with live vaccines [146]. Both the United States and the United Kingdom have focused attention on the development of subunit vaccines based on the fraction 1 (F1) capsular antigen and the low calcium response protein V (LcrV) antigens [123,147,148] (Figure 2). As discussed earlier, *Y. pestis* expresses a capsule antigen F1, encoded by the *caf1* gene, which has been shown to have significant antiphagocytic activity [51,149,150,151,152,153]. LcrV is a secreted virulence protein which is essential for survival in the host, acting as an immunosuppressive factor [65]. Early work demonstrated a protective effect associated with active vaccination with V fractions [154,155,156,157]. Several groups reported that anti-V antibodies may be protective by promoting phagocytosis of bacteria and reducing bacteria-induced cytotoxicity [158,159,160]. In addition to being a protective antigen, LcrV was later shown to be a multi-factorial protein that is an important structural component of the T3SS (injectisome) and a secreted protein that is trafficked into eukaryotic cells; furthermore, the LcrV antigen can regulate aspects of the host immune response extracellularly by induction of anti-inflammatory IL-10 [65,66,67,68,69,70,71,72,73,74,75,76,156]. Unfortunately, important differences have been documented between LcrV proteins isolated from various *Y. pestis* strains which may hamper protective efficacy of subunit vaccines that rely on a specific polymorph for induction of immunological protection but may have a limited cross-reactive response [161]. Nevertheless, both F1 and V have been studied as components of subunit vaccines and have been shown to confer significant protection against bubonic and pneumonic plague induced by encapsulated strains of *Y. pestis,* with few documented concerns about tolerability or safety [155,157,162,163,164,165].

The primary subunit vaccine candidate in the United States is the recombinant F1-V, a fusion protein of the F1 capsular antigen and the *lcrV* gene product [166,167,168,169,170]. The United Kingdom pursued a similar strategy for vaccination but retained the F1 and V immunogens as separate proteins [171,172,173]. PharmAthene further advanced this concept and developed a recombinant dual antigen vaccine for plague, composed of rF1 + rV, named RypVax™ [174,175]. A truncated LcrV antigen, rV10, developed by Schneewind and colleagues in 2011 was also assessed [176]. Both vaccines, rF1 + rV and rV10, were tested and demonstrated efficacy against pneumonic plague infection in mice, guinea pigs and Cynomolgus macaques [176,177] but not in African green monkeys [125]. Further investigations of enhancing immunogenicity or delivery of these subunit vaccines have been attempted or are ongoing by several groups [178,179,180,181,182,183,184,185].

The two-component vaccines have been variably effective and are vulnerable to breakthrough infection. Researchers found that the combination of these subunits significantly enhances protection against bubonic and pneumonic plague in different animal models [176,186,187]. The protection afforded by this vaccine strategy against F1-negative strains relies entirely on the LcrV antigen component of the F1-V fusion protein. Since there is evidence for V heterogeneity within Yersinia species, the potential exists that naturally occurring or engineered strains harboring altered LcrV antigens could overcome F1-V-induced immunity [161].

Other vaccine strategies have attempted to identify vaccine antigens that would protect animals against non-encapsulated strains. Andrews demonstrated that active vaccination against YopD could protect mice against an F1-negative strain of *Y. pestis* [188]. Later studies by Ivanov et al. further characterized the protective efficacy of YopD and created fusion proteins of YopBD and YopBDE [189]. Ivanov et al. achieved protection with both active and passive vaccination strategies using these proteins, but these data suggested that anti-Yop immune responses were most protective against non-encapsulated *Y. pestis* [189]. While there has been progress in vaccine development, the need remains for other reliably protective and readily deployable countermeasures against plague.

## 6. Antibodies

There has been significant interest in and effort towards identifying antibody therapies in animal models of plague. In 1963, Lawton and coworkers identified protection associated with anti-LcrV polyclonal serum passively administered to mice. Importantly, it was determined that the protection associated with this polyclonal serum could not be completely attributable to the LcrV antigen due to the likelihood of other Yersinia proteins present in the material used to generate the sera [190]. Later, Motin et al. demonstrated with more modern techniques that a highly purified preparation of the V protein could be produced and used for polyclonal sera generation [190]. These anti-sera directed against native LcrV antigen or recombinant LcrV antigens were able to provide substantial passive protection against *Y. pestis* infection.

Table 1 and Table 2 and the following paragraphs provide summary information extracted from noteworthy publications describing particularly effective monoclonal antibodies in mouse models of plague. Hill et al. demonstrated that a monoclonal antibody directed against the LcrV could protect against a fully virulent strain of *Y. pestis* and went on to demonstrate which regions of the LcrV antigen appeared to be protective epitopes [156]. Quenee et al. and Amemiya et al. further elaborated on the importance of the specific binding sites on protective anti-LcrV monoclonal antibodies [191,192]. The mAb 7.3 was shown to protect macrophages from *Y. pestis*-induced cell death and also to promote phagocytosis of the bacteria [158,193]. Importantly, much of the protection afforded by mAb 7.3 can be attributed to the blocking of the T3SS machinery. When anti-V antibodies block this injectisome, the immunomodulation and anti-phagocytic Yop effectors are not efficiently secreted, thus blocking cytotoxic phenotypes and promoting phagocytosis of the bacterial cells [158]. This concept was further supported by Eisele and Anderson when they demonstrated that both blocking the T3SS as well as inducing phagocytosis are required from an anti-LcrV mAb in order to optimally protect against pneumonic plague in a mouse model of disease [194]. Novel Adenovirus-mediated delivery has also been used to demonstrate the protection associated with anti-LcrV antigen mAbs [195].

Partly due to the known heterogeneity amongst LcrV protein sequences from different bacterial isolates [161,196,197,198], a multi-antigen strategy was postulated to be required to optimize protection. Anderson et al. also demonstrated that it was possible to passively protect mice against either bubonic or pneumonic plague [199] with anti-F1 antibodies. These anti-F1 monoclonal antibodies, however, would not protect against F1-negative (non-encapsulated) strains of *Y. pestis* (Figure 2). Since immunity to F1 is not sufficient to protect in all cases, there was renewed focus on the incorporation of the LcrV antigen or other proteins for multifactorial strategies.

Combinations of F1 and LcrV monoclonal antibodies were shown to protect mice against both bubonic and pneumonic plague either prophylactically or as post-exposure therapy [200,201]. More recent efforts have continued to examine strategies combining anti-F1 and anti-LcrV antibodies [202]. Xiao et al. identified three human mAbs: m252 for anti-F1, and m253 and m254 for anti-LcrV. They found that anti-F1 human mAb (m252) provided better protection in mice than anti-LcrV human mAbs. However, an apparent synergistic effect was found when they combined all three antibodies. Another three anti-F1 (F5C10, F6E5, and F2H5) mAbs were tested against subcutaneous challenge with *Y. pestis* 141 and showed different protection levels. Liu et al. demonstrated that mAb F2H5 from a mouse hybridoma provides complete protection from bubonic plague in BALB/c mice [203]. Altogether, it would be feasible that these mAbs against F1 or LcrV can be utilized as a potential treatment or prophylaxis for humans against plague. Additional anti-*Y. pestis* human antibodies have recently been produced and are currently being evaluated for therapeutic application [204].

## 7. Advantages of Monoclonal Antibodies (mAbs) and Final Perspectives

Currently, there are only a few mAbs licensed for use against bacterial infectious agents. To date, mAb against *Clostridium difficile* and *Bacillus anthracis* toxins are licensed in Europe and the USA [205,206,207]. Other work has focused on mAbs against *Staphylococcus aureus* or *Pseudomonas aeruginosa* [208]. The renewal of interest in mAb therapies is even more relevant given the current era of worrisome multidrug resistance in bacterial isolates of clinical importance. Several clinically relevant bacteria such as *Burkholderia pseudomallei* and *Burkholderia mallei* are naturally resistant to many antibiotics [209]. Examples of emerging MDR bacteria include but are not limited to *S. aureus*, *C. difficile*, *P. aeruginosa*, *Acinetobacter baumeii*, *Enterococci*, etc [210,211,212,213]. This MDR trait has been observed with *Y. pestis,* and the lack of effective therapies for such an infection could result in catastrophic loss of life, whether from a naturally emerging outbreak or an intentional attack utilizing purposefully engineered bacteria.

Recent strategies have focused on nimble and rapid platforms that can be leveraged when combatting outbreaks and emerging threats. Advances in rapid mAb discovery, optimization, and production make this an attractive strategy that could significantly decrease the time required to get a product to civilian populations in the midst of an outbreak or to military personnel deployed to areas where endemic disease could be an issue and an effective vaccine has yet to be identified. Other more novel strategies involve administering nucleic acids that encode the protective mAb to patients. In that case, a nucleic acid encoding the immunoglobulin may be manufactured or obtained from a cDNA library or nucleic acid isolated from any tissue or cells expressing the antibody. These nucleic acids can be cloned into a suitable vector and administered into an infected patient [202].

These concepts have been useful recently during West African Ebola virus outbreaks [214]. In this epidemic, mAbs were demonstrated to be important novel treatment strategies. Products such as ZMapp® and others were critical in the response to the Ebola virus epidemic [214]. Viruses of recent or continual concern including HIV, Zika, and COVID-19 have also been targets of these novel strategies [215,216,217,218,219,220]. Monoclonal antibodies will likely be of the utmost importance moving forward when developing novel medical countermeasures against existing and rapidly emerging infectious diseases.

## Figures and Tables

**Table 1:** Monoclonal antibodies shown to protect mice in bubonic plague models (subcutaneous challenge with bacteria).

Antigen ^a^	mAb Description	Antibody/Therapeutic Administration			*Y. pestis* Challenge Strain ^d^	Challenge Dose (LD_50_)	Ref.
		Route	Concentration	Schedule			
F1; LcrV; F1V + LcrV	human IgG1	IP	500 µg	24 h pre–120 h post	CO92	25–40	202
LcrV	mouse	IP	350 µg	24 h pre	GB	12	156
LcrV ^b^	mouse IgG1, IgG2a	IP	1–500 µg	24 h pre	CO92 or C12	21–39	192
LcrV; F1 + LcrV	mouse IgG1	IP	0.7–100 µg	4 h pre–96 post	GB	9.6–91,000	200
LcrV	mouse IgG1	IP	200 µg	1 h pre	CO92	20	191
F1 ^c^	mouse IgG1	IP	125–500 µg	6 h or 24 h pre	CO92	48–54	199
F1	not reported	IV	100 µg	24 h pre	141	600	203

a, unless otherwise noted mAbs tested in BALB/c female mice; b, tested in BALB/c and Swiss Webster mice; c, tested in Swiss Webster female mice; d, bacteria delivered via subcutaneous injection.

**Table 2:** Monoclonal antibodies shown to protect mice in pneumonic plague models (intranasal instillation or exposure to aerosolized bacteria).

Antigen ^a^	mAb Description	Antibody/Therapeutic Administration			*Y. pestis* Challenge Strain ^f^	Challenge Dose (LD_50_)	Ref.
		Route	Concentration	Schedule			
LcrV	mouse IgG1	IP	35 µg	4 h pre–96 h post	GB	88	156
LcrV ^b^	mouse IgG1	IP	200 or 400 µg	1 h pre	CO92 ^g^	15–20	194
LcrV ^c^	mouse IgG2b	IP, IV	100–500 µg, 10^11^ pu ^e^	94 h pre–24 h post	CO92 ^g^	363–9080	195
F1 ^d^	mouse IgG1	IP	125–500 µg	6 h or 24 h pre	CO92	29–74	199
F1 + LcrV	mouse IgG1	IT	77.5 µg (each)	2 h post	GB	27	200

a, unless otherwise noted mAbs tested in BALB/c female mice; b, tested in C57BL/6 female mice; c, tested in BALB/c female and C57BL/6 male mice; d, tested in Swiss Webster female mice; e, antibodies delivered as hybridoma supernatant or via adenovirus vector; f, unless otherwise noted aerosolized bacteria were delivered; g, intranasal instillation was used for challenge
